# Investigating the Effects of Olaparib on the Susceptibility of Glioblastoma Multiforme Tumour Cells to Natural Killer Cell-Mediated Responses

**DOI:** 10.3390/pharmaceutics15020360

**Published:** 2023-01-20

**Authors:** Jennifer Moran, Eimear Mylod, Laura E. Kane, Caroline Marion, Emily Keenan, Marianna Mekhaeil, Joanne Lysaght, Kumlesh K. Dev, Jacintha O’Sullivan, Melissa J. Conroy

**Affiliations:** 1 Cancer Immunology Research Group, Department of Physiology, Trinity College Dublin, D02 R590 Dublin, Ireland; 2 Cancer Immunology and Immunotherapy Group, Department of Surgery, Trinity Translational Medicine Institute and Trinity St. James’s Cancer Institute, St. James’s Hospital, Trinity College Dublin, D08 W9RT Dublin, Ireland; 3 Department of Surgery, Trinity Translational Medicine Institute and Trinity St. James’s Cancer Institute, St. James’s Hospital, Trinity College Dublin, D08 W9RT Dublin, Ireland; 4 Drug Development Research Group, Department of Physiology, School of Medicine, Trinity College Dublin, D02 R590 Dublin, Ireland

**Keywords:** NK cells, Glioblastoma multiforme, immunotherapy, Olaparib, PARP Inhibitor

## Abstract

Glioblastoma multiforme (GBM) is the most common adult primary brain malignancy, with dismal survival rates of ~14.6 months. The current standard-of-care consists of surgical resection and chemoradiotherapy, however the treatment response is limited by factors such as tumour heterogeneity, treatment resistance, the blood–brain barrier, and immunosuppression. Several immunotherapies have undergone clinical development for GBM but demonstrated inadequate efficacy, yet future combinatorial approaches are likely to hold more promise. Olaparib is FDA-approved for BRCA-mutated advanced ovarian and breast cancer, and clinical studies have revealed its utility as a safe and efficacious radio- and chemo-sensitiser in GBM. The ability of Olaparib to enhance natural killer (NK) cell-mediated responses has been reported in prostate, breast, and lung cancer. This study examined its potential combination with NK cell therapies in GBM by firstly investigating the susceptibility of the GBM cell line T98G to NK cells and, secondly, examining whether Olaparib can sensitise T98G cells to NK cell-mediated responses. Here, we characterise the NK receptor ligand profile of T98G cells and demonstrate that Olaparib does not dampen T98G susceptibility to NK cells or elicit immunomodulatory effects on the function of NK cells. This study provides novel insights into the potential combination of Olaparib with NK cell therapies for GBM.

## 1. Introduction

Glioblastoma multiforme (GBM) is a highly invasive and aggressive primary brain malignancy with a dismal 5-year survival rate of less than 7% [1] The median progression-free survival of patients with this devastating and incurable disease stands at ~7 months, while overall survival is ~14.6 months post-diagnosis [2]. The current standard-of-care consists of an intensive trimodal regimen of surgical resection followed by adjuvant Temozolomide chemotherapy and radiotherapy; however, resistance to the standard-of-care remains a significant challenge in GBM, as approximately 80% of patients experience a relapse [2,3].

Natural killer (NK) cells are crucial tumour-killing immune cells whose dysfunction in cancer is associated with poorer patient outcomes [4,5,6]. As such, they are emerging as valuable players in the cellular therapy space [7,8], offering an attractive “off-the-shelf” alternative to T cells as they do not require antigen specificity and can be sourced from allogeneic blood, cell lines, or stem cells [9,10]. The activation of NK cells is balanced by the ligation of their activating and inhibitory NK receptors (NKRs) with their NKR ligands on target cells [11].

As immunotherapies emerge as the fourth pillar of cancer treatment, there is opportunity for cellular-based immunotherapies to significantly advance this therapeutic space. A plethora of trials are ongoing assessing the utility of immunotherapies such as immune checkpoint inhibitors (ICI), a peptide/dendritic cell vaccination, and T cell therapies in the treatment of GBM; however, they have so far failed to demonstrate any significant clinical benefit [6]. The disappointing efficacy in GBM is likely due to the immunosuppressive and immune evasive tumour microenvironment (TME), which is influenced by suppressive factors such as Transforming Growth Factor-β (TGF-β), hypoxia, lactate, Prostaglandin E2 (PGE2), Indoleamine 2,3-dioxygenase (IDO), and adenosine [12,13,14,15,16,17]. IDO can directly reduce the activation of NK cells in the TME and promote the accumulation of regulatory T cells (Treg) in GBM tumours which go on to secrete immunosuppressive cytokines and inhibitory proteins to further dampen NK cells’ functions [13,18,19]. PGE2 reduces the expression of NK cell-activating receptors and inhibits IFN-γ production and NK cell proliferation, while TGF-β drives NK cell metabolic dysfunction, inhibits the secretion of perforin, and reduces the expression of NKG2D and DNAM-1 receptors [12,14,20,21]. The proteolytic shedding of NKR ligands from the tumour cell’s surface by metalloproteases also significantly diminishes NK cell-mediated immunity in GBM [22]. The proteolytic cleavage of NKR ligands, such as MICA/B, generates soluble ligands which can induce endocytosis and the subsequent degradation of their cognate receptor, NKG2D on NK cells, impairing the function of NK cells within the GBM TME [23,24]. As such, future combinatorial approaches to overcome immunosuppression and immune modulation within the TME are likely to hold more promise for the treatment of GBM patients.

The NK cell infiltration of GBM tumours correlates with patient survival, thus supporting the development of NK cell therapies for this poor prognosis malignancy [25,26,27]. It has also been suggested that the loss of NK cells plays a role in the progression of low-grade gliomas to malignant high-grade tumours [27]. Off-the shelf NK cell-based therapies are showing significant promise for the treatment of GBM, however, the highly immunosuppressive GBM TME presents a significant challenge for their success [15,16,28]. We propose that this may be overcome by identifying and targeting the immunosuppressive components of GBM tumours using combination therapies and genetically engineered NK cells. For instance, NK cell therapies engineered with dominant negative TGF-β receptors retain their anti-tumour functions in the presence of TGF-β in GBM [20]. In addition, there are numerous ongoing preclinical and clinical studies exploring the effects of anti-TGF-β strategies in combination with anti-cancer therapies, highlighting the potential of combinatorial therapies in immunosuppressive solid tumours [29,30,31,32].

PARP inhibitors are small molecule inhibitors which interfere with base excision DNA repair and induce genomic instability via the polyADP-ribosylation of target proteins [33]. Inhibitors of PARP1/2 are FDA-approved for the treatment of tumours with germline mutations in double strand break repair genes, such as BRCA-mutated breast and ovarian cancer [34]. Olaparib is a PARP1/2 inhibitor which has already shown promise in GBM as a safe and effective chemo- and radio-sensitiser; however, little is known about its immunomodulatory effects [35,36,37]. Previous studies have demonstrated that Olaparib can enhance NK cell-mediated responses across several cancer types [34,38]. The ability of Olaparib to upregulate death receptor expression has been reported in GBM, acute myeloid leukaemia (AML), lung, and ovarian cancer, and this resulted in a lower apoptotic threshold and increased apoptosis [38,39]. Furthermore, Olaparib sensitises prostate carcinoma cells to antibody-dependent cellular cytotoxicity (ADCC) [34]. Furthermore, the phase I OPARATIC study demonstrated that the penetrative capacity of clinically relative concentrations of Olaparib into GBM tumours reflects that of breast tumours, demonstrating that the systemic delivery of Olaparib in GBM would not be hindered by the blood–brain barrier [40].

This study elucidated the susceptibility of GBM tumour cells to NK cells and NK cell therapies by profiling the NKR ligand surface expression of T98G cells and examining their NKR ligand shedding. In addition, the potential combination of Olaparib and NK cell therapy was investigated by first examining the efficacy of Olaparib to sensitise T98G cells to NK cell-mediated responses and, second, elucidating the potential off-target immunomodulatory effects of a systemic PARP inhibition on NK cell phenotype and function.

## 2. Materials and Methods

### 2.1. Analysis of Relationship between NKR Ligand Expression and Disease-Free and Overall Survival of GBM Patients

Information on the disease-free and overall survival of GBM patients was downloaded from the cBioPortal website [41,42]. The combined cohort included 2212 patients from 7 studies (Mayo Clinic, 2019; CPTAC, Cell 2021; Columbia, Nat Med. 2019; TCGA, Cell 2013; TCGA, Nature 2008; TCGA, Firehose Legacy; TCGA, PanCancer Atlas). Details on the tumour grade were unavailable for this cohort, though it is understood from the corresponding manuscripts of the included studies that these patients represented a mixed population of cancer stages. This cohort was searched independently for patients with capped relative linear copy number values for each gene of interest. Patients with available data for the ligands of interest were divided into quartiles based on their capped relative linear copy number values and assessed for significance using a Logrank test from the cBioPortal software. As these copy number values were generated via high-throughput sequencing methods, it was not necessary to control for batch effects across these data. Indeed, a recent systematic review and meta-analysis of 250 studies showed that the variability introduced by patient-to-patient comparisons is much larger than any variability that could be introduced by the different high-throughput platforms used across these studies [43]. Significant differences in the survival between the quartiles are noted where the Logrank test *p*-value <0.05.

### 2.2. Cell Culture of T98G Cells

The T98G Glioblastoma multiforme cell line was obtained from the American Type Culture Collection. T98G are fibroblast-like cells derived from a male GBM patient. T98G cells are resistant to standard-of-care temozolomide [44]. The T98G cells were cultured in DMEM/F12 supplemented with 10% foetal bovine serum (FBS) and 1% penicillin–streptomycin (complete DMEM/F12(cDMEM/F12), Gibco, Waltham, MA, USA) at 37 °C in a humidified atmosphere of 5% CO_2_. The cells were routinely mycoplasma tested.

### 2.3. Identifying the Non-Lethal Dose of Olaparib in T98G Cells Using Cell Counting Kit (CCK) 8 Assay

To identify the non-lethal dose of Olaparib, T98G cells were seeded at a density of 10,000 cells per 100 µL of cDMEM/F12 and treated with 0.1 μM, 1 μM, 5 μM, 10 μM, 20 μM, 50 μM, or 100 μM of Olaparib (SelleckChem, Houston, TX, USA) for 24 h. The CCK8 assay was performed as per the manufacturer’s instructions (Sigma, St Louis, MO, USA). In brief, the cell media was removed and replaced following 24 h drug treatments and 5 μL of CCK8 was added to each well. The plate was incubated at 37 °C for 2.5 h and read every 45 min at 450 nm on a Glomax platereader (Promega, Madison, WI, USA).

### 2.4. Quantifying the Effects of Olaparib on NKR Ligand Expression by T98G Cells

T98G cells were seeded at a density of 50,000 cells per 500 μL of cDMEM/F12 and treated for 24 h with non-lethal doses of Olaparib (5 µM and 10 µM), as determined by a CCK8 assay. A total of 5 μM and 10 μM were shown to be optimal non-lethal doses in other GBM cell lines, primary cultures, and GBM tissue samples [38,45,46]. Following treatment, the supernatants were collected and cryopreserved at −80 °C. The cells were trypsinised and stained for their flow cytometry using the following antibodies for NKR-activating ligands, PVRL2-PE (Miltenyi Biotec, Bergisch Gladbach, Germany), B7-H6-PE (R&D, Minneapolis, MN, USA), 4-1BBL-PerCP-Cyanine5.5 (BioLegend, San Diego, CA, USA), ULBP3-APC (BioLegend), PVR-FITC (BioLegend), MICA/B-PE-Cyanine7 (BioLegend), NKR inhibitory ligands, HLA-E-PerCP-Cyanine5.5 (BioLegend), PD-L1-PE-Cyanine7 (BioLegend), and death receptors, Fas-FITC (BioLegend) and TRAIL-R2-PE (BioLegend). All samples were acquired using the BD FACS CANTO II (BD Biosciences, Franklin Lakes, NJ, USA) flow cytometer and analysed using FlowJo v10 software (BD Biosciences). The gating strategies are available in Supplementary Figure S1.

### 2.5. Assessing the Effects of Olaparib on NK Cell Phenotype

Peripheral blood mononuclear cells (PBMC) were isolated from the blood of healthy donors by density gradient centrifugation and resuspended in RPMI supplemented with 10% FBS and 1% penicillin–streptomycin (complete RPMI (cRPMI), Gibco). PBMC were seeded at a density of 500,000 cells per 500 µL of cRPMI and treated for 24 h with 5 μM or 10 μM of Olaparib. Following treatment, the cells were surface stained for flow cytometric analysis using CD56-FITC (BioLegend), CD3-APC-Cyanine7 (BioLegend), and the following antibodies for activating NKRs, NKp46-PE-Cyanine7 (BioLegend), DNAM-1-PE (BioLegend), NKp30-BV421 (BioLegend), NKG2D-PE-Cyanine5 (BioLegend), CD16-PE-Cyanine7 (BioLegend), inhibitory receptors NKG2A-APC (Miltenyi Biotec), PD-1-PE-Cyanine7 (BioLegend), and TIGIT-PerCP-Cyanine5.5 (BioLegend), death receptor ligands TRAIL-APC (BioLegend) and FasL-BV421 (BioLegend), and phenotypic markers, CD57-PE (BioLegend), CD69-BV510 (BioLegend), and CD27-Pacific blue (BioLegend). All samples were acquired using the BD FACS CANTO II (BD Biosciences) flow cytometer and analysed using FlowJo v10 software (BD Biosciences). NK cells were defined as CD56^+^ CD3^−^ in the lymphocyte gate. The gating strategies are available in Supplementary Figure S1.

### 2.6. Quantifying the Immunomodulatory Effects of T98G Cells and the Potential Rescuing Effects of Olaparib on NK Cell Phenotype and Function

For culture in T98G cell supernatant, PBMC were seeded at a density of 1 × 106 cell per 1ml and cultured in media alone (cDMEM/F12) or with 50% T98G cell supernatant for 24 h. Following co-culture, PBMC were surface stained for flow cytometry using CD56-FITC (Miltenyi Biotec), NKG2D-PE-Cyanine5, NKp30-BV421, and CD3-APC-Cyanine7 (BioLegend).

For co-culture, the T98G cells were trypsinised at 80% confluency and seeded at a density of 500,000 cells per 1 mL of cDMEM/F12 media. PBMC were co-cultured at a 1:2.5 ratio with T98G cells with or without 5 µM or 10 µM of Olaparib treatment for 24 h. Following co-culture, PBMC were surface stained for flow cytometry using CD56-FITC (BioLegend), CD3-APC-Cyanine7 (BioLegend), and the following antibodies for activating NKRs, NKp46-PE-Cyanine7 (BioLegend), DNAM-1-PE (BioLegend), NKp30-BV421 (BioLegend), NKG2D-PE-Cyanine5 (BioLegend), and CD16-PE-Cyanine7 (BioLegend), inhibitory receptors NKG2A-APC (Miltenyi Biotec), PD-1-PE-Cyanine7 (BioLegend), and TIGIT-PerCP-Cyanine5.5 (BioLegend), death receptor ligands, TRAIL-APC (BioLegend) and FasL-BV421 (BioLegend), and phenotypic markers, CD57-PE (BioLegend), CD69-BV510 (BioLegend), and CD27-Pacific blue (BioLegend). For intracellular staining, the cells were stimulated with 10 ng/mL of PMA and 1 μg/mL of Ionomycin for four hours (Sigma). CD107a-PE (BD Biosciences) was added to detect degranulation. After one hour, 10 μg/mL of Brefeldin A (Sigma) was added for the remaining three hours. The cells were stained for the surface markers CD56-FITC and CD3-APC-Cyanine7 (BioLegend). The cells were stained for the intracellular markers with Granzyme B-PE-Cyanine5, TNF-α-APC, IL-10-BV421, and IFN-γ-BV510 (BioLegend) using the FIX&PERM Cell Fixation and Permeabilization Kit (Nordic MUBio, Susteren, The Netherlands). All samples were acquired using the BD FACS CANTO II (BD Biosciences) flow cytometer and analysed using FlowJo v10 software (BD Biosciences). NK cells were defined as CD56^+^ CD3^−^ in the lymphocyte gate. The gating strategies are available in Supplementary Figure S1.

### 2.7. Quantification of Cytokine Secretion and NKR Ligand Shedding on T98G Cells

Supernatants from Olaparib-treated T98G cells were collected and cryopreserved at −80 °C. Human IL-6 duo-set ELISA, human B7-H6 duo-set ELISA, human ULBP3 duo-set ELISA (R&D), human MICA/B ELISA, human TIMP-1 ELISA, human PGE2 ELISA, and human TGF-β ELISA (Assay Genie, Dublin, Ireland) were carried out as per the manufacturer’s instructions. The absorbance values were measured at 595 nm using a VersaMax plate reader and SoftmaxPro 6.1 software, from which a standard curve was generated to deduce the total protein concentrations.

### 2.8. Assessing the Effects of Olaparib on NK Cell Chemotaxis towards T98G Cells

PBMC were isolated from non-cancer donors and were resuspended at 1 × 106 cells per ml of cRPMI and treated with 5 μM or 10 μM of Olaparib (SelleckChem) for 24 h. The cells were subsequently added at a density of 0.2 × 106 cells/100 µL RPMI to a 5 µm pore transwell filter system (CellQart, Northeim, Germany). To assess the effects of Olaparib on the NK cell migratory capacity, the migration of Olaparib-treated PBMC towards untreated T98G cell supernatant was quantified. To assess the effects of Olaparib on the chemotactic potential of the GBM tumour secretome, the migration of untreated PBMC towards the supernatants of 5 μM or 10 μM of Olaparib-treated T98G cells was measured. The transwell system was incubated for 2 h at 37 °C, 5% CO_2_. The cells were collected from the lower chamber and stained for flow cytometric analysis with CD56-FITC and CD3-APC-Cyanine7 (BioLegend). CountBright beads (Invitrogen, Waltham, MA, USA) were used to enumerate the migrated CD56^+^ CD3^−^ NK cells. Cells were acquired using a BD FACS CANTO II (BD Biosciences) flow cytometer and analysed using FlowJo v10 software (BD Biosciences).

### 2.9. Statistical Analysis

Statistical analysis was performed using GraphPad Prism version 9 (GraphPad software, San Diego, CA, USA). The statistical significance between groups was determined by a Wilcoxon matched-pairs signed rank test or one-way ANOVA with post hoc Dunnett’s or Tukey’s test as appropriate. *p* < 0.05 was considered to be the significance threshold.

## 3. Results

### 3.1. Low Copy Numbers of Genes Encoding Activating NKR Ligands and Death Receptors Is Associated with Poorer Survival in GBM Patients

**Figure 1:**
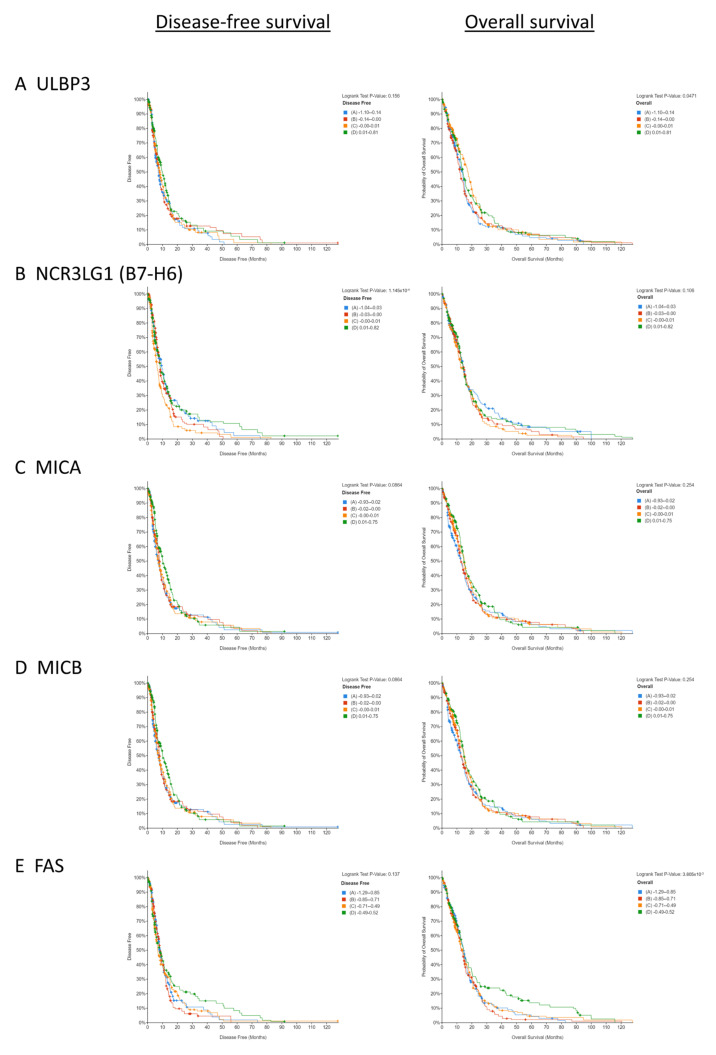

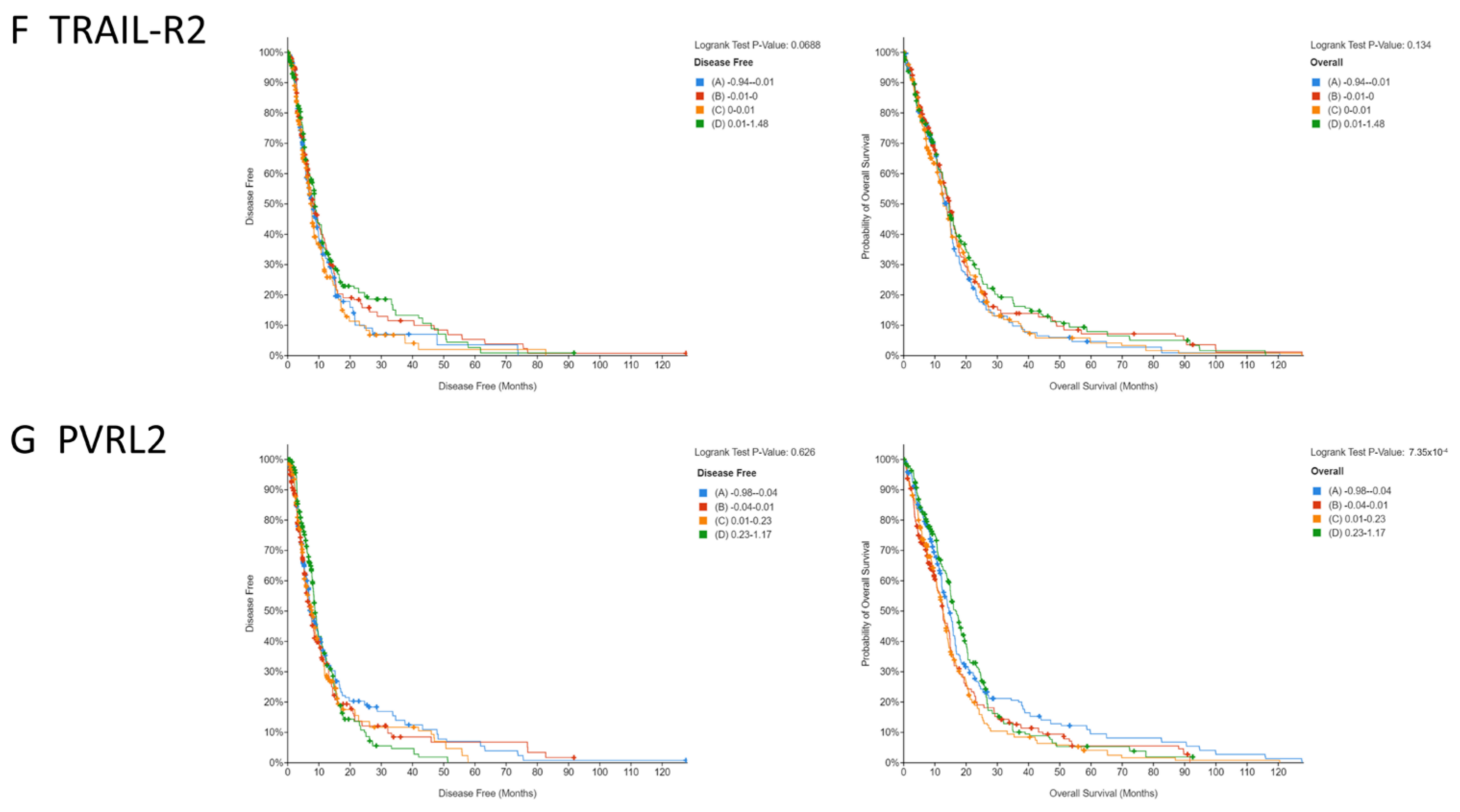
Low copy number of genes encoding NK cell-activating receptor ligands and death receptors is associated with a poorer survival in GBM. Kaplan–Meier’s graphs showing the association between capped relative linear copy number values and (left) disease-free survival and (right) overall survival and expression of (**A**) ULBP3, (**B**) NCR3LG1, (**C**) MICA, (**D**) MICB, (**E**) FAS, (**F**) TRAIL-R2, and (**G**) PVRL2. Patients with available data were divided into quartiles based on their capped relative linear copy number values and assessed for significance using a Logrank test.

Analysis of the GBM patient data from cBioPortal revealed that a low copy number of the NKR ligand gene ULBP3 (*p* = 0.0471) and the death receptor gene FAS (*p* = 0.003805) was associated with a poorer overall survival in GBM patients (Figure 1A,E). In contrast, a high copy number of dual-activating and inhibitory ligand PVRL2 (*p* = 0.0007354) was associated with a poorer overall survival in GBM patients (Figure 1G). When disease-free survival was evaluated as an endpoint in this GBM patient cohort, a low copy number of the NKR ligand gene NCR3LG1 (B7-H6) (*p* = 0.00001145) was associated with a poorer disease-free survival (Figure 1B). While a statistical significance was not reached, the trends in these data also indicated that a low copy number of the genes encoding NKR ligands MICA (*p* = 0.0864) and MICB (*p* = 0.0864), and death receptor TRAIL-R2 (*p* = 0.0688), were associated with a poorer disease-free survival in GBM patients (Figure 1C,D,F).

### 3.2. Olaparib Alters the Surface Expression of Death Receptors and NKR Ligands on T98G Cells

**Figure 2:**
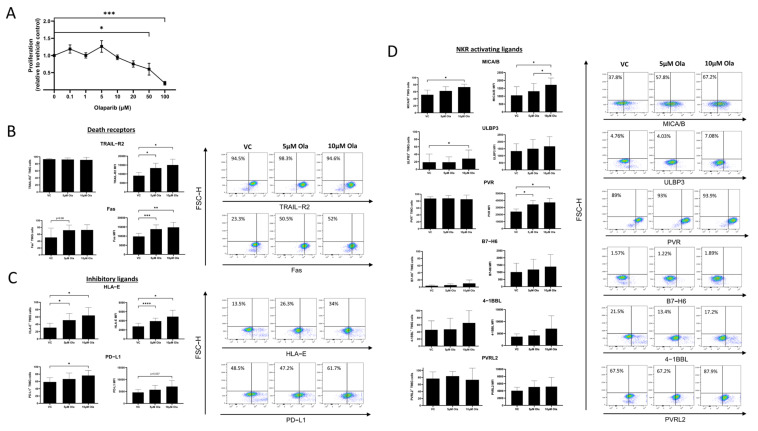
Olaparib treatment significantly alters natural killer receptor ligand surface expression and death receptor expression by T98G cells. (**A**) Line graph showing the proliferation of T98G cells following treatment with 0.1 μM, 1 μM, 5 μM, 10 μM, 20 μM, 50 μM, and 100 μM of Olaparib for 24 h (*n* = 4). (**B**) (Left) Bar charts showing the MFI and % frequencies of T98G cells positive for death receptor expression following treatment with 5 μM or 10 μM of Olaparib for 24 h [TRAIL-R2 (top, *n* = 4); Fas (bottom, *n* = 5)]. (Right) Representative dot plots showing % frequencies of death receptor-expressing T98G cells following treatment with 5 μM or 10 μM of Olaparib for 24 h. (**C**) (Left) Bar charts showing the MFI and % frequencies of T98G cells positive for inhibitory ligand expression following treatment with 5 μM or 10 μM of Olaparib for 24 h [HLA-E (top, *n* = 4); PD-L1 (bottom, *n* = 5)]. (Right) Representative dot plots showing % frequencies of inhibitory receptor-expressing T98G cells following treatment with 5 μM or 10 μM of Olaparib for 24 h. (**D**) (Left) Bar charts showing the MFI and % frequencies of T98G cells positive for NKR-activating ligand expression following treatment with 5 μM or 10 μM of Olaparib for 24 h [MICA/B, ULBP3, B7-H6 (*n* = 5); PVR, 4-1BBL, PVRL2 (*n* = 4)]. (Right) Representative dot plots showing % frequencies of NKR-activating ligand-expressing T98G cells following treatment with 5 μM or 10 μM of Olaparib for 24 h. * *p* < 0.05, ** *p* < 0.01, *** *p* < 0.001, **** *p* < 0.0001 by one-way ANOVA with post hoc Dunn’s test.

We first sought to identify non-lethal doses of Olaparib to examine as part of this study. There were no significant differences in T98G proliferation following treatment with 0.1 μM, 1 μM, 5 μM, 10 μM, or 20 μM of Olaparib for 24 h (Figure 2A). There was a significant decrease in T98G proliferation following a treatment with 50 μM or 100 μM of Olaparib for 24 h; VC vs, 50 μM (1 vs. 0.6, *p* = 0.04), VC vs. 100 μM (1 vs. 0.19, *p* = 0.0003) We chose 5 μM and 10 μM of Olaparib for this study in order to obtain the most potent immunoregulatory effects (Figure 2A).

To further interrogate the susceptibility of GBM tumours to NK cells, the NKR and death receptor profiles of T98G cells were assessed and the impact of Olaparib treatment on such profiles was investigated. The expression of death receptors TRAIL-R2 (*n* = 4) and Fas (*n* = 5) measured by the mean fluorescence intensity (MFI) was significantly higher on T98G cells treated with 5 µM and 10 µM of Olaparib, compared to the cells treated with vehicle control DMSO (VC); TRAIL-R2; VC vs. 5 µM of Ola (9052. 75 vs. 13,311, *p* = 0.0131), VC vs. 10 µM of Ola (9052.75 vs. 1502.38, *p* = 0.016), Fas; VC vs. 5 µM of Ola (9805.8 vs. 13,796.1, *p* = 0.001), VC vs. 10 µM of Ola (9805.8 vs. 14,769, *p* = 0.0078) (Figure 2B). There were no significant differences in the basally high frequencies of TRAIL^+^ T98G cells (*n* = 4) following treatment with Olaparib for 24 h, while frequencies of Fas^+^ T98G cells (*n* = 5) were trending higher following a treatment with 5 µM and 10 µM of Olaparib compared to cells treated with VC; VC vs. 5 µM of Ola (45.75% vs. 69.7%, *p* = 0.09), VC vs. 10 µM of Ola (45.75% vs. 73.95%, *p* = 0.09) (Figure 2B).

Interestingly, frequencies of activating receptor-expressing MICA/B^+^ (*n* = 5) and ULBP3^+^ (*n* = 5) T98G cells were significantly higher following a treatment with 10 µM of Olaparib compared to a treatment with VC; MICA/B VC vs. 10 µM of Ola (51.44% vs. 73.46%, *p* = 0.01), ULBP3 VC vs. 10 µM of Ola (18.826% vs. 28.2%, *p* = 0.04). The MFIs of MICA/B^+^ and PVR^+^ T98G cells were also significantly higher following a treatment with Olaparib compared to VC; MICA/B; VC vs. 10 µM of Ola (1051.2 vs. 1718, *p* = 0.04), 5 µM of Ola vs. 10 µM of Ola (1312.6 vs. 1718, *p* = 0.02), PVR; VC vs. 5 µM of Ola (24,200 vs. 34,459, *p* = 0.0175), VC vs. 10 µM of Ola (24,200 vs. 37,172, *p* = 0.0106) (Figure 2D). There were no significant differences in the frequencies or MFI of PVRL2^+^, 4-1BBL^+^, or B7-H6^+^ T98G cells (*n* = 4) following a treatment with Olaparib (Figure 2D).

The frequencies of immune checkpoint-expressing and inhibitory receptor-bearing T98G cells were also increased following Olaparib treatment. Frequencies of PD-L1^+^ (*n* = 5) and HLA-E^+^ (*n* = 4) T98G cells were significantly higher following a treatment with 5 µM and 10 µM of Olaparib compared to VC; PD-L1; VC vs. 10 µM of Ola (58.5% vs. 76.28%, *p* = 0.03), HLA-E; VC vs. 5 µM of Ola (30.8% vs. 51.2%, *p* = 0.01), VC vs. 10 µM of Ola (30.8% vs. 64.05%, *p* = 0.02) (Figure 2C). Expression levels of HLA-E (*n* = 4) measured by MFI were also significantly higher in T98G cells following a treatment with 5 µM or 10 µM of Olaparib compared to VC; VC vs. 5 µM of Ola (2757.75 vs. 3920, *p* < 0.0001), VC vs. 10 µM of Ola (2757.75 vs. 4902.2, *p* = 0.02) (Figure 2C).

### 3.3. Direct Co-Culture or Exposure to the Soluble Environment of T98G Cells Diminished NK Cell-Activating Receptor Expression

**Figure 3:**
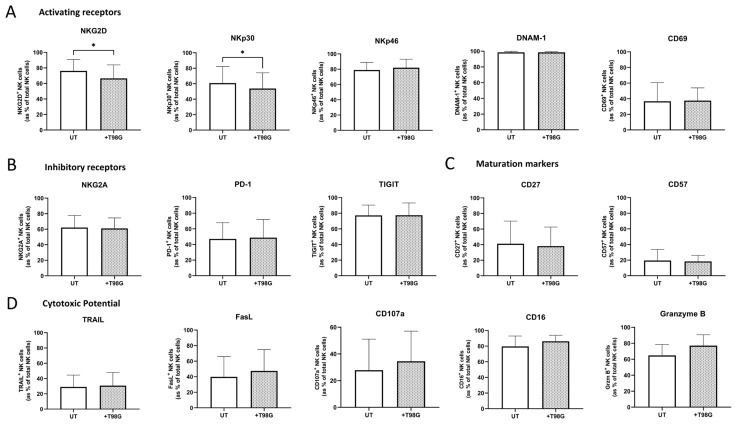

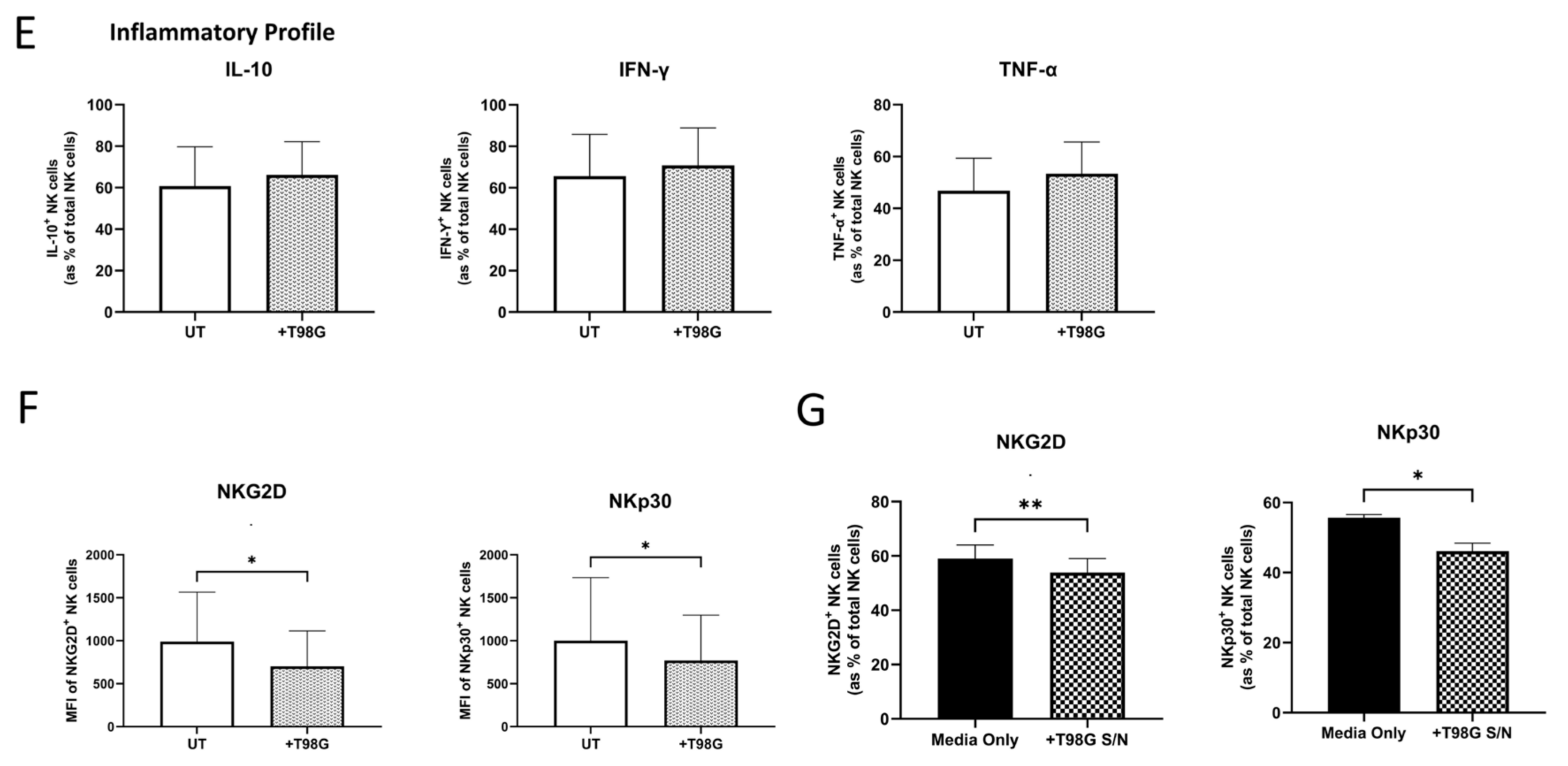
Co-culture with T98G cells suppresses NKG2D and NKp30 surface expression on healthy donor-derived NK cells in vitro. Bar charts showing frequencies of (**A**) NKG2D^+^, NKP30^+^, NKp46^+^, DNAM-1^+^, CD69^+^ (activating receptors), (**B**) NKG2A^+^, PD-1^+^, TIGIT^+^ (inhibitory receptors), (**C**) CD27^+^, CD57^+^ (maturation markers), (**D**) TRAIL^+^, FasL^+^, CD107a^+^, CD16^+^, Granzyme B^+^ (cytotoxic markers), (**E**) IL-10^+^, IFN-γ^+^, TNF-α^+^ (inflammatory markers) (*n* = 6) [DNAM-1, CD16 *n* = 3] NK cells as a % of total NK cells following co-culture alone (untreated/UT, white) or with T98G cells (+T98G, pattern) for 24 h. (**F**) Bar charts showing the MFI of NKG2D^+^ and NKP30^+^ NK cells following co-culture alone (untreated/UT, white) or with T98G cells (+T98G, pattern). (**G**) Bar charts showing frequencies of NKG2D^+^ and NKP30^+^ NK cells as a % of total NK cells following culture in media only (black) or with T98G supernatant (+T98G S/N, pattern). * *p* < 0.05, ** *p* < 0.01 by Wilcoxon test.

Since the reinvigoration of the NK cell responses in GBM is likely to be beneficial to GBM patients, it is prudent to elucidate the impact of the GBM TME on infiltrating NK cells and NK cell therapies. Here, healthy donor-derived NK cells were co-cultured with T98G cells or exposed to T98G supernatants for 24 h to determine the immunomodulatory challenges facing NK cells and NK cell therapies within the GBM TME. There were significantly lower frequencies of NKG2D^+^ and NKp30^+^ NK cells following the culture with the T98G cell supernatant (S/N) compared to the NK cells cultured with media only for 24 h (both *n* = 3); NKG2D; media only vs. +T98G S/N (59% vs. 53%, *p* = 0.0062), NKp30; media only vs. +T98G S/N (55.6% vs. 46.1%, *p* = 0.02) (Figure 3G). In line with the diminished activated phenotype of NK cells exposed to the soluble GBM TME, there were significantly lower frequencies of NKG2D^+^ and NKp30^+^ NK cells following a co-culture with T98G cells compared to the NK cells cultured alone (untreated (UT)) for 24 h (both *n* = 6); NKG2D; UT vs. +T98G (76.1% vs. 66.5%, *p* = 0.0313), NKp30; UT vs. +T98G (60.75% vs. 53.80%, *p* = 0.0313) (Figure 3A). Furthermore, the MFI of NKG2D^+^ and NKp30^+^ NK cells was significantly lower following a co-culture with T98G cells compared to the NK cells cultured alone (untreated (UT)) for 24 h (both *n* = 6); NKG2D; UT vs. +T98G (989.5 vs. 702, *p* = 0.0313), NKp30; UT vs. +T98G (1000 vs. 796, *p* = 0.0313) (Figure 3F).

Frequencies of NKp46^+^, DNAM-1^+^, CD69^+^, NKG2A^+^, PD-1^+^, TIGIT^+^, TRAIL^+^, FasL^+^, CD107a^+^, CD16^+^, Granzyme B^+^, CD27^+^, and CD57^+^ NK cells remained unaltered following 24 h of co-culture with T98G cells (all *n* = 6) (Figure 3A–D). Furthermore, such 24 h of co-culture did not alter the inflammatory profile of NK cells (*n* = 6) (Figure 3E).

### 3.4. Concurrent Olaparib Treatment and Direct T98G Co-Culture Did Not Alter NK Cell Phenotype or Function

**Figure 4:**
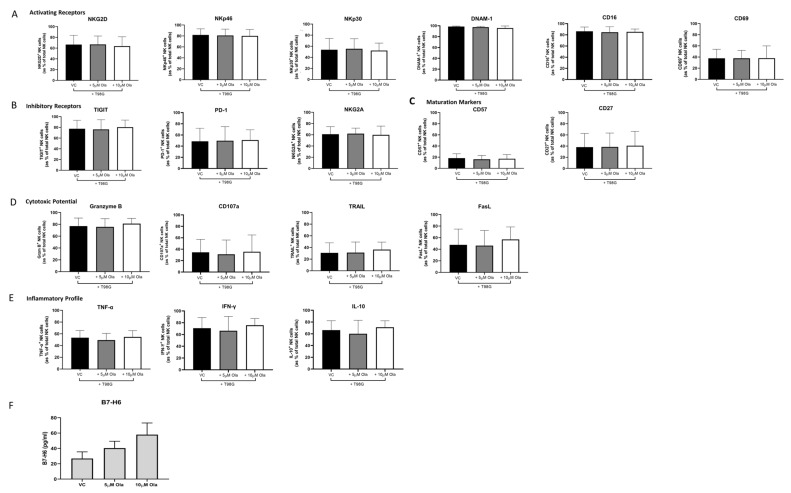
Concurrent Olaparib treatment and co-culture with T98G cells did not alter the phenotype, cytotoxic potential, or inflammatory profile of healthy donor-derived NK cells. Bar chart showing % frequencies of NK cells expressing (**A**) NKG2D, NKp46, NKp30, DNAM-1 (activating receptors), (**B**) TIGIT, PD-1, NKG2A (inhibitory receptors) (**C**) CD57, CD27, CD69, CD16 (maturation markers), (**D**) TRAIL, FasL, CD107a, Granzyme B (cytotoxic markers), and (**E**) TNF-α, IFN-γ, IL-10 (inflammatory markers) as % of total NK cells, following co-culture with T98G cells (black) and treatment with 5 μM (grey) or 10 μM (white) of Olaparib for 24 h (all *n* = 6) [DNAM-1, CD16 *n* = 3]. (**F**) Bar chart showing soluble B7-H6 in the secretome of T98G cells treated with vehicle control (VC) or 5 µM or 10 µM of Olaparib for 24 h (*n* = 3). One-way ANOVA with post hoc Dunn’s test.

Olaparib treatment of the NK:T98G cell co-cultures resulted in no significant differences in NKG2D^+^ and NKp30^+^ NK cells (Figure 4A). In fact, the frequencies of all activating and inhibitory receptor-expressing NK cells were sustained following a concurrent treatment with Olaparib and co-culture with T98G cells (Figure 4A,B). Furthermore, Olaparib treatment of the NK:T98G cell co-cultures resulted in no significant differences in the cytotoxic potential, inflammatory profile, or maturation phenotype of NK cells (*n* = 6, Figure 4C–E). In line with its inability to rescue the dampened NKG2D and NKp30 expression by the T98G-exposed NK cells, Olaparib did not significantly attenuate B7-H6 shedding by T98G cells (*n* = 3) (Figure 4F).

### 3.5. Olaparib Treatment Elicits no Significant Effects on the Phenotype, Inflammatory Profile, Cytotoxic Potential or Migratory Capacity of NK Cells

**Figure 5:**
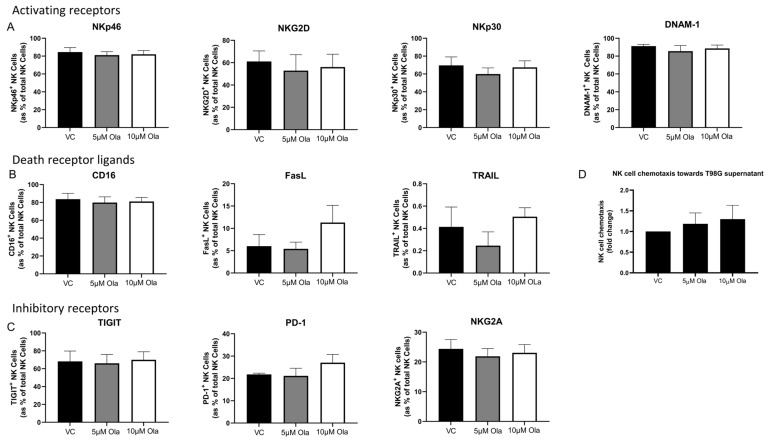
NK cell phenotype is not significantly altered by Olaparib treatment. Bar charts showing frequencies of (**A**) NKp46^+^, NKG2D^+^, NKp30^+^ (activating receptors), (**B**) CD16^+^, FasL^+^, TRAIL^+^ (death receptor ligands) and (**C**) TIGIT^+^, PD-1^+^, NKG2A^+^ inhibitory receptor NK cells as % of total NK cells following treatment with vehicle control (black) or 5 µM (grey) or 10 µM (white) of Olaparib for 24 h. (**D**) Bar chart showing the migration of NK cell towards the soluble chemotactic cues of T98G cells following 24 h treatment of NK cells with 5 µM or 10 µM of Olaparib, relative to vehicle control (VC). One-way ANOVA with post hoc Dunn’s test.

While Olaparib does not significantly alter the function of NK cells within the context of the GBM TME, it is crucial to ascertain whether it elicits systemic effects on circulating NK cells. To address this, healthy donor-derived NK cells were treated with non-lethal doses of Olaparib and their function, phenotype, and ability to migrated towards soluble cues in the GBM TME were assessed. There were no significant differences in the frequencies of NKp46^+^, NKG2D^+^, NKp30^+^, DNAM-1^+^, CD16^+^, FasL^+^, TRAIL^+^, TIGIT^+^, PD-1^+^, or NKG2A^+^ NK cells following a treatment with 5 µM or 10 µM of Olaparib for 24 h relative to the NK cells treated with VC (all *n* = 4) (Figure 5A–C). Furthermore, Olaparib treatment did not alter the migratory capacity of NK cells towards the soluble chemotactic factors of T98G cells (*n* = 5) (Figure 5D).

### 3.6. Olaparib Treatment Enhanced IL-6 Secretion by T98G Cells but Did Not Negatively Affect Chemoattraction of NK Cells

**Figure 6:**
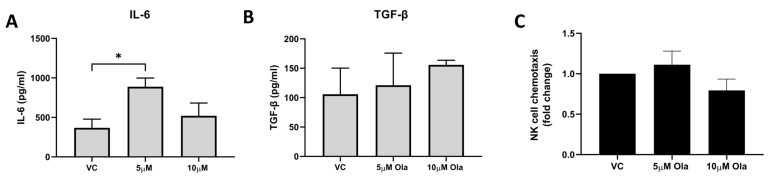
Olaparib treatment significantly altered the secretome of T98G cells in vitro but did not alter their chemoattraction of NK cells. Bar charts showing the levels of soluble (**A**) IL-6 (*n* = 7) and (**B**) TGF-β (*n* = 3) in the secretome of T98G cells treated with vehicle control (VC) or 5 µM or 10 µM of Olaparib for 24 h. (**C**) Bar chart showing NK cell chemotaxis towards supernatants of T98G cells previously treated with vehicle control (VC), 5 µM or 10 µM of Olaparib for 24 h (*n* = 5). * *p* < 0.05, by one-way ANOVA with post hoc Dunn’s test.

While Olaparib altered the NKR ligand surface profile of T98G cells, it did not significantly alter the NKR ligand shedding. Here, we investigated whether Olaparib treatment impacted the inflammatory secretome of GBM tumour cells and their chemotactic cues. Olaparib significantly increased the secretion of IL-6 by T98G cells (*n* = 7); VC vs. 5 µM of Ola (368.5 pg/mL vs. 888.3 pg/mL, *p* = 0.0276) (Figure 6A). However, Olaparib did not significantly alter the secretion of TGF-β by T98G cells (*n* = 3) (Figure 6B). Importantly for a successful NK cell infiltration of the tumour, Olaparib treatment did not negatively alter the chemoattraction of NK cells by the T98G cells (Figure 6C). The levels of soluble PGE2 and TIMP-1 protein were undetectable in the cell line supernatant from the T98G cells treated with Olaparib.

## 4. Discussion

NK cells and their diverse array of surface receptors hold a crucial role in cancer immunosurveillance and the expression of such NKRs and their corresponding ligands on tumour cells is critical to the successful eradication of GBM [47,48,49,50,51]. NK cell immunotherapy is a novel intervention that has demonstrated great potential for success in GBM, a malignancy with dismal survival rates and no curative treatment options [52,53]. The efficacy of adoptively transferred NK cells has been demonstrated in a variety of different cancers, including GBM [54,55]. Furthermore, an NK cell therapy using human placental hematopoietic stem cell-derived NK cells (CYNK-001) has recently gained fast track designation from the FDA for recurrent GBM, further highlighting the extraordinary potential of NK cells as a therapeutic tool for this poor prognosis cancer [56,57].

Previous studies support the hypothesis that Olaparib may augment the immune landscape of GBM, thereby improving the recognition and killing of tumour cells by NK cells [38,39,58,59,60]. There is compelling evidence that PARP inhibitors such as Olaparib increase the death receptor expression in various solid tumours, sensitising them to TRAIL-mediated apoptosis [38,39,58]. In addition, several studies have demonstrated that PARP inhibition may upregulate the expression of ligands of the activating receptor NKG2D on tumour cells via the induction of genotoxic stress and stalled DNA replication forks [59,60]. There are a number of NKRs beyond TRAIL and NKG2D and their cognate ligands, which have not been investigated in the context of GBM, and there is a paucity of data on the effects of Olaparib in this space. Here, for the first time, we have characterised the NKR ligand expression profile of GBM tumour cells, interrogated the associations between NKR ligand expression and GBM patient survival, and investigated the effects of Olaparib on this profile. In addition, we have performed a thorough investigation of the off-target immunomodulatory effects of Olaparib on NK cells within and outside the GBM TME.

There is a lack of consensus on the links between NKR ligand expression and prognosis in a host of cancers [61,62,63,64,65]. In ovarian, head and neck, and pancreatic cancers, the expression of NKR ligands B7-H6, ULBP2, RAET1E, and death receptor TRAIL-R were associated with a poorer prognosis [61,63,64]. However, in the setting of colorectal and breast cancers, high NKR ligand MICA/B and ULBP2 expression was associated with a better prognosis for patients [62,65]. As such, we first sought to interrogate publicly available gene datasets to ascertain the association between NKR ligands and GBM patient survival. Our analysis uncovered that a low copy number of genes encoding NK-activating receptor ligands ULBP3 and B7-H6, and death receptor FAS, is associated with a poorer overall survival in GBM patients. These data support the hypothesis that NK cell dysfunction contributes to poorer outcomes in GBM and that NK cell therapies may offer survival benefits in this malignancy. Given the positive effects of Olaparib on death receptor expression and NK cell-mediated responses in other solid cancers, we hypothesised that it might augment GBM tumour susceptibility to NK cells.

To elucidate the effects of Olaparib on GBM cells and its potential to potentiate NK cell-mediated responses in the GBM microenvironment, we first assessed changes in NKR ligand expression on the surface of the GBM cell line T98G cells in response to PARP inhibition. Given that the genomic data analyses in this study were performed on NKR ligand and death receptor gene expression and not the functional protein expressed on the surface of the cancer cell, it was first critical to extensively assess the NKR ligand surface expression profile of GBM cells. Our study revealed the unique NKR ligand and death receptor profile of T98G cells and demonstrated that Olaparib treatment increased the expression of the ligands for the activating receptors NKG2D;ULBP3, and MICA/B. This is in line with a previous study in AML where treatment with Olaparib upregulated the expression of NKG2D ligands on the surface of AML cells [66]. Previous studies have linked PARP inhibition and NKG2D ligand upregulation on tumour cells via the induction of genotoxic stress and stalled DNA replication forks [59,60].

Previous work by Karpel-Massler et al. and Meng et al. reported that PARP inhibition induced the upregulation of death receptors TRAIL-R2 and Fas on various tumour cells [38,39]. The data presented here demonstrated that Olaparib significantly increased the expression of TRAIL-R2 and Fas on the surface of T98G cells. It is important to note that almost all T98G cells express TRAIL-R2 at the baseline and Olaparib treatment altered the level of expression on the cell surface, thus potentially strengthening the activation signal to GBM tumour-infiltrating NK cells. Given the association identified between the low copy number of ULBP3 and Fas and a poor survival in GBM patients, their increased expression on T98G cells following Olaparib treatment indicates a potential new mechanism through which PARP inhibition may confer clinical benefits in GBM patients. In contrast to the high expression levels of death receptors on GBM cells in response to Olaparib treatment, the expression levels of their corresponding ligands, TRAIL and FasL, on NK cells were relatively low and were unaffected by Olaparib treatment and co-culture with T98G cells. The death receptor ligand expression is regulated by cytokines, such as IFN-γ, and the effects of Olaparib may be further boosted by cytokine stimulation of NK cells. IFN-γ has been shown to induce TRAIL expression on NK cells and Zhu et al. demonstrated that IFN-γ signalling is amplified in GBM [67,68]. The cytokine IL-12 is a potent inducer of IFN-γ that stimulates NK cell proliferation and cytotoxicity, increases TRAIL expression and suppresses metastasis, and therefore, may be considered in combination with an NK cell-based therapy [69,70]. Death receptor-mediated killing is a crucial mechanism by which NK cells eradicate tumour cells and this study has further established that PARP inhibition consistently upregulates the expression of death receptor TRAIL-R2 on tumour cells and may have utility in combination with immunotherapies such as TRAIL-R2 agonists, which are already in trials in pancreatic and colorectal cancer (NCT03082209). Furthermore, for the first time, we have shown that death receptor Fas is upregulated on GBM cells in response to Olaparib. These data suggest that a bimodal approach of a TRAIL-R2 agonist and PARP inhibition may be beneficial for GBM patients.

The solid TME is an overall immunosuppressive environment primed to dampen and dysregulate the functionality of NK cells [16,28,52,71,72]. Hypoxia, nutrient deprivation, and the acidic pH coupled with NKR ligand shedding and the overabundance of immunosuppressive cytokines, such as TGF-β and PGE2, make the GBM TME a hostile environment for NK cells [16,28,71,73]. We therefore sought to investigate the impact of a direct co-culture of T98G and NK cells on NK cell phenotype and function. Our data revealed that T98G cells could significantly modulate the surface expression of activating NKRs on primary human NK cells. The frequencies and MFI of NKG2D^+^ and NKp30^+^ NK cells were significantly lower following co-culture with T98G cells or their supernatants. A shift in the NKR profile of NK cells has been reported following exposure to oesophageal adenocarcinoma (OAC) and ovarian cancer cells and is known to dampen the surveillance and cytotoxic activity of NK cells [22,74]. As such, we next sought to identify potential mediators of such reductions. Here, we report abundant levels of TGF-β but no detectable PGE2 in the T98G cell secretome. Previous reports have found a role for TGF-β in downregulating the expression of activating receptors NKp30 and NKG2D on the surface of NK cells, including in glioma patients [22,75]. This presents targeting the secretion of TGF-β as a central therapeutic approach in GBM to limit immune dysfunction. The methods to target TGF-β include pan-TGF-β neutralising antibodies, antisense TGF-β oligonucleotides, and TGF-βR1 inhibitors; however, the results from trials in GBM patients have been disappointing and further highlight the importance of targeting multiple pathways to derive a clinical benefit in GBM patients [76].

The basal surface expression of the NKR ligand B7-H6 was low on T98G cells and was not significantly altered by Olaparib treatment. NKR ligand shedding is a well described mechanism of tumour immune evasion whereby the cleavage of membrane-bound ligands by matrix metalloproteinases results in soluble ligands in the TME which can interact with receptor-bearing NK cells [24,77]. This results in the defective expression of activating receptors and, therefore, facilitates the tumour cell evasion of NK cell-mediated immune surveillance. In OAC, hepatocellular carcinoma, ovarian carcinoma, and neuroblastoma, links between elevated soluble B7-H6 and NK cell dysfunction have been established [22,78,79,80]. Here, we report detectable levels of soluble B7-H6 in the GBM cell secretome, suggesting that soluble B7-H6 in the GBM TME may also contribute to the diminished NKp30 expression on the surface of NK cells following co-culture with T98G cells or their soluble environment. Of note, we did not detect soluble MICA/B or ULBP3 by ELISA, in line with a previous study in OAC [22]. There is evidence to suggest that ULBP3 is shed into exosomes in contrast to the established enzymatic shedding mechanism of other NKR ligands by metalloproteases, which may account for the undetectable ULBP3 levels in the GBM supernatant [81,82]. In addition, B7-H6 has been shown to modulate the downregulation of activating receptors NKG2D and NKp46, indicative of its effects beyond its own receptor, suggesting that exposure to T98G cell-derived B7-H6 may at least contribute to the diminished NKG2D+ NK cell frequencies observed in this study. This highlights the potential of targeting NKR ligand shedding with therapeutic intent to dampen the suppression of NK cells and this has already been shown as a promising approach [83]. A host of ADAM inhibitors have been explored both pre-clinically and clinically; however, initial positive results have yet to translate into a clinical benefit. As such, more specific inhibitors are in development with the aim of limiting the side effects in patients [84].

Interestingly, a low-dose but not high-dose treatment with Olaparib resulted in increased levels of the cytokine IL-6 in the GBM secretome, suggesting that low-dose Olaparib can contribute to the inflammatory TME. IL-6 is a pleiotropic cytokine which has heterogeneous effects in GBM, promoting cancer cell survival, metastasis, and invasion [85]. Furthermore, IL-6 can impair NK cell cytotoxicity via STAT3 signalling [86]. However, here we report no significant impairments in Granzyme B or CD107a expression by NK cells when co-cultured with T98G and treated with Olaparib. Indeed, the data presented here suggest that co-culture of T98G cells and NK cells concurrently with Olaparib treatment did not alter the markers of NK cell cytotoxicity, maturation, or cytokine production. These findings provide an insight into the timing of Olaparib administration with respect to an NK cell therapy and suggest that the exposure of T98G cells to Olaparib prior to the introduction of NK cells may better facilitate enhanced NK cell activation by allowing time for Olaparib-induced alterations in the NKR ligand and death receptor profiles of GBM tumours. The sequenced timings of immunotherapy as part of a combination therapeutic regimen are critical to ensure that NK cell functionality is maintained, and our data indicate that Olaparib would elicit the most optimal results if administered prior to an NK cell therapy, priming the TME for NK cell attack.

Overall, co-culture with T98G cells did not hamper NK cell functionality, with markers of cytokine production being unaffected. Furthermore, Olaparib treatment alone did not significantly alter NK cell phenotype and function, suggesting that Olaparib elicits greater effects on NK cell target cells and thus is effective to use alongside an NK cell-based therapy. A previous study has shown a role for PARP-1 in NK cell migration to the site of viral infections [87]. As such, we sought to explore whether the inhibition of PARP would hinder NK cell migration towards the soluble chemotactic cues of T98G cells. Here, we report no significant alterations in NK cell migration, suggesting that systemic PARP inhibition will not impede NK cell trafficking to the tumour. Furthermore, Olaparib treatment does not appear to alter the chemotactic cues of the T98G cell secretome to the extent that it impacts NK cell migration.

Olaparib treatment induced a significant increase in the frequency of HLA-E^+^ and PD-L1^+^ T98G cells as well as the cell surface expression of HLA-E at 24 h. This could have a myriad of ramifications for an NK cell therapy in GBM as both HLA-E and PD-L1 are inhibitory ligands. HLA-E is a non-classical HLA class I molecule that binds to KIRs and NKG2 receptor family members such as NKG2A to regulate NK cell function [88]. We identified that the corresponding receptor NKG2A was expressed on NK cells and was unchanged by Olaparib treatment with and without co-culture with T98G. HLA-E is typically expressed on normal healthy cells to protect them from NK cell-mediated killing and this system can be manipulated in cancer to the advantage of the tumour [89]. Anti-NKG2A targeting antibodies are in development and could complement the inhibition of PARP along with an NK cell therapy to boost their effector function [90]. Increased PD-L1 expression with Olaparib treatment of GBM cells is in line with a previous study in breast cancer [33]. However, increased PD-L1 expression provides an opportunity for synergy between PARP inhibitors and ICI, both of which are rapidly establishing their place as significant treatment modalities for numerous cancer types [91,92]. The corresponding immune checkpoint receptor PD-1 was found to be moderately expressed on NK cells and was not increased by co-culture or Olaparib treatment, indicating that PD-L1 is the optimal target for an ICI in this context. There is compelling preclinical evidence for synergy between PARP inhibitors and ICIs and the efficacy of this dual treatment combination is under clinical investigation in breast and ovarian cancer patients [93]. Furthermore, a combination blockade of both NKG2A and PD-1 is currently being evaluated in clinical trials and shows promise in this context in colorectal cancer (NCT02671435) [7]. Based on these findings, we hypothesise that a trimodal combination of an anti-PD-L1/NKG2A antibody, PARP inhibitors, and an NK cell therapy may exude synergistic effects in GBM.

In this study, we report that low activating NKR ligand and death receptor expression is associated with lower survival in GBM patients, providing a justification for the potentiation of NK cell-mediated responses in this cancer. For the first time, we have identified that Olaparib treatment can enhance the surface expression of such activating NKR ligands and death receptors on T98G cells, thus identifying a novel mechanism of action through which Olaparib elicits clinical benefits in GBM. Moreover, these data suggest that treatment with Olaparib prior to the introduction of an NK cell-based therapy would be optimal for this combined approach in GBM. Furthermore, while GBM tumour cells may modulate NKR-activating receptor expression by NK cells, Olaparib treatment elicited no detrimental effects on NK cell phenotype or function. Overall, we propose that Olaparib treatment elicits beneficial effects on GBM tumour susceptibility to NK cells and NK cell therapies, and this combination could offer clinical utility to improve GBM patient survival.

## Data Availability

Data available on request.

## Supplementary Materials

The following supporting information can be downloaded at: https://www.mdpi.com/article/10.3390/pharmaceutics15020360/s1, Figure S1: Gating Schemes for T98G Cells and NK Cells.
