# Supplementary material for: Investigating the Effects of Olaparib on the Susceptibility of Glioblastoma Multiforme Tumour Cells to Natural Killer Cell-Mediated Responses
Source: Pharmaceutics. 2023 Jan 20;15(2):360. doi: 10.3390/pharmaceutics15020360 (PMC9959685; doi:10.3390/pharmaceutics15020360)
Supplement: Supplementary file 1 [file pharmaceutics-15-00360-s001.zip › pharmaceutics-2133927-supplementary.pdf]

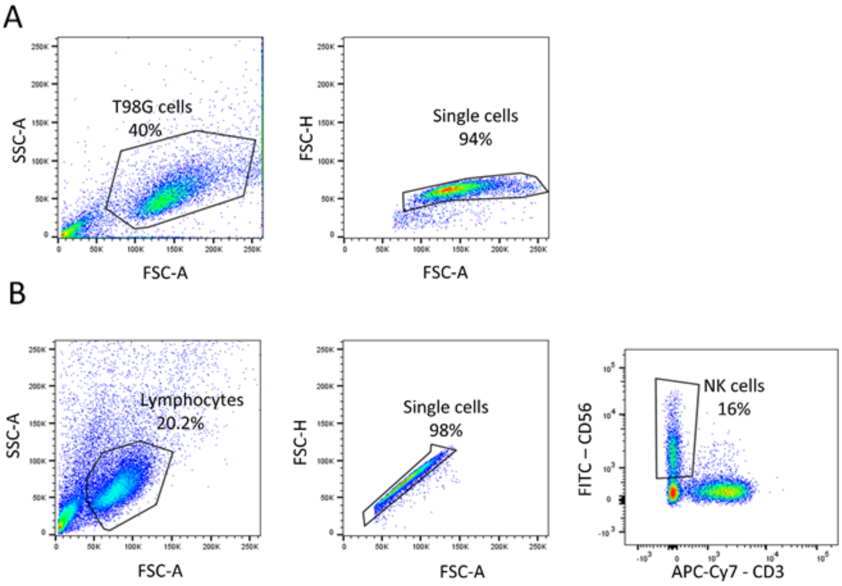

Supplementary Figure S1: Gating Schemes for T98G Cells and NK Cells. A (Left-Right): Gate 1 (T98G cells) included all cells in the FSC-A versus SSC-A plot. Gate 2 (Single cells) excluded doublet cells using the FSC-H versus FSC-A plot. **B (Left-Right)** Gate 1 (Lymphocytes) included all lymphoid cells based on size and granularity using the FSC-A versus SSC-A plot. Gate 2 (Single cells) excluded doublet cells using the FSC-H versus FSC-A plot. Gate 3(NK cells) included only cells which were $CD56^{+}$ and $CD3^{-}$.
